# Can 3 mg·kg^−1^ of Caffeine Be Used as An Effective Nutritional Supplement to Enhance the Effects of Resistance Training in Rugby Union Players?

**DOI:** 10.3390/nu13103367

**Published:** 2021-09-25

**Authors:** Ryan A. Tamilio, Neil D. Clarke, Michael J. Duncan, Rhys Morris, Jozo Grgic, Jason Tallis

**Affiliations:** 1 Centre for Applied Biological and Exercise Sciences, Alison Gingell Building, Coventry University, Priory Street, Coventry CV 15FB, UK; tamilior@cucollege.coventry.ac.uk (R.A.T.); ab1633@coventry.ac.uk (N.D.C.); aa8396@coventry.ac.uk (M.J.D.); ac9669@coventry.ac.uk (R.M.); 2 Institute for Health and Sport, Victoria University, Melbourne, VIC 8001, Australia; jozo.grgic@live.vu.edu.au

**Keywords:** resistance exercise, strength, power, ergogenic aids, performance

## Abstract

The present study uniquely examined the effect of 3 mg·kg^−1^ chronic caffeine consumption on training adaptations induced by 7-weeks resistance training and assessed the potential for habituation to caffeine’s ergogenicity. Thirty non-specifically resistance-trained university standard male rugby union players (age (years): 20 ± 2; height (cm): 181 ± 7; body mass (kg): 92 ± 17) completed the study), who were moderate habitual caffeine consumers (118 ± 110 mg), completed the study. Using a within-subject double-blind, placebo-controlled experimental design, the acute effects of caffeine intake on upper and lower limb maximal voluntary concentric and eccentric torque were measured using isokinetic dynamometry (IKD) prior to and immediately following a resistance training intervention. Participants were split into strength-matched groups and completed a resistance-training program for seven weeks, consuming either caffeine or a placebo before each session. Irrespective of group, acute caffeine consumption improved peak eccentric torque of the elbow extensors (*p* < 0.013), peak concentric torque of the elbow flexors (*p* < 0.005), total eccentric work of the elbow flexors (*p* < 0.003), total concentric work of the knee extensors (*p* < 0.001), and total concentric and eccentric work of the knee flexors (*p* < 0.046) following repeated maximal voluntary contractions. Many of these acute caffeine effects were still prevalent following chronic exposure to caffeine throughout the intervention. The training intervention resulted in significant improvements in upper and lower body one-repetition maximum strength (*p* < 0.001). For the most part, the effect of the training intervention was equivalent in both the caffeine and placebo groups, despite a small but significant increase (*p* < 0.037) in the total work performed in the participants that consumed caffeine across the course of the intervention. These results infer that caffeine may be beneficial to evoke acute improvements in muscular strength, with acute effects prevalent following chronic exposure to the experimental dose. However, individuals that consumed caffeine during the intervention did not elicit superior post-intervention training- induced adaptations in muscular strength.

## 1. Introduction

A wealth of scientific evidence, summarised in a recent umbrella review [1], documents small but significant improvements in aerobic endurance [2], anaerobic power [2,3], muscular endurance and strength [4,5], and sport-specific skills [6] following acute caffeine ingestion. Given caffeine’s widespread performance-enhancing benefits, it is a popular ingredient in many commercially available products targeted to augment adaptions evoked by exercise training. Most previous research has focused on acute caffeine effects on a single exercise bout [2,7,8]. However, there is a dearth of evidence examining if such acute benefits applied over the long-term can manifest in enhanced, chronic adaptations to exercise training.

Several meta-analyses have reported an ergogenic effect of acute caffeine ingestion for muscular strength [1,3,5]. Due to these acute effects, it would seem intuitive that improved performance in a bout of resistance exercise multiplied over the duration of a resistance training regime may evoke an elevated training response [9]. However, this assumption has not been thoroughly explored and the potential beneficial response of caffeine during chronic exposure may be offset by habituation to its effects. Although a point of contention [10,11,12] there is evidence to suggest that chronic consumption of 3 mg·kg^−1^ of caffeine in low habitual caffeine users (<75 mg/day^−1^) results in intolerance after 4 weeks [13]. Furthermore, in some cases, acute effects of caffeine on exercise performance are not demonstrated in high habitual users (426 mg/day) [14]. Direct comparisons are challenging given the lack of standardised thresholds for determining high and low habitual caffeine use [15]. However, confounding ideas surrounding the chronic use of caffeine for exercise performance would appear to work in paradox, inciting a need for further investigation.

Only two studies examined if chronic caffeine ingestion can augment resistance training adaptations. In resistance-trained males, Kemp et al. [9] demonstrated that 3 mg·kg^−1^ of caffeine consumed before every exercise session resulted in superior improvements in the bench press and squat one repetition maximum (1RM) compared to the placebo group following 6 weeks of resistance training. Using the same dose and a similar population, recent work by Giráldez-Costas et al. [16] indicated no significant difference in training-induced adaptions in bench press 1RM between groups ingesting either caffeine or placebo during a 4-week resistance training intervention. However, the group ingesting caffeine had a more pronounced improvement in movement velocity when assessed across the force-velocity spectrum.

The disparity in caffeine-induced improvements in maximal strength may be related to the difference in the training regimes and duration of the program. Furthermore, previous studies did not account for baseline strength when assigning participants to training groups, which may have influenced the results. Given the limited research in the area and the inconclusive findings, further investigation is required. Whilst these initial studies offer important insight, work is now needed to understand if chronic caffeine consumption during resistance training induces regional and contractile mode-specific changes given that caffeine’s effect on strength may differ between concentric and eccentric models of activity, contractile velocity, and specific muscle groups [17,18,19,20]. Furthermore, previous work fails to consider intersessional performance between caffeine and placebo groups across the course of the training intervention, which is an important driver to enhance resistance-training adaptation. Moreover, previous studies also fail to consider the potential impact of habituation, which may limit the proposed benefits of chronic caffeine supplementation. For this to be achieved, a more complex within and between subject’s experimental design is needed.

Given the outlined important gaps in the literature, the aims of the present study were threefold: (1) using a within-subjects experimental design the present work sought to assess the acute effect of 3 mg·kg^−1^ of caffeine consumption on the maximal voluntary concentric and eccentric strength of the knee and elbow flexors and extensors in a population of male rugby union players; (2) using a between-subjects experimental design to assess the effects of 3 mg·kg^−1^ of caffeine consumption on adaptations to a 7-week resistance training program in participants matched for baseline strength; (3) using a within-subjects experimental design to reassess the acute effect of 3 mg·kg^−1^ of caffeine consumption on the maximal voluntary concentric and eccentric strength of the knee and elbow flexors and extensors post completion of the training regime. As such, the present work provides the most detailed examination of the effects of chronic caffeine consumption on regional and contractile mode-specific adaptations to resistance training and uniquely considers the impact of caffeine habituation in inhibiting augmentation of the training response. It was hypothesised that (1) acute caffeine consumption would evoke improved muscular strength, which would manifest in a small but significant superior training adaptations; (2) that the group that consumed caffeine during the training intervention would demonstrate a reduced acute effect of caffeine on measures of muscular strength following the intervention.

## 2. Materials and Methods

### 2.1. Participants

Following ethics approval from Coventry University (reference code; P76381; approved on 3/12/2018) and informed consent, 40 apparently healthy participants from the Coventry University Men’s rugby union team agreed to participate in the study. The current study was also in compliance with the declaration of Helsinki [21]. Participants trained with the team twice per week and played competitively once per week. This continued throughout the duration of the study. Potential participants were excluded if they were: suffering from a musculoskeletal injury that prevented safe completion of the exercise trials; were consuming psychoactive medication; had any other underlying contradictions to exercise; or habitually consumed high levels of caffeine (i.e., >6.00 mg/ kg/day) [15]. Participants completed a health screen questionnaire prior to each visit. Over the course of the investigation, 10 participants dropped out due to injury (*n* = 3) (not related to the experimental protocol), illness (*n* = 2), or for reasons not stated (*n* = 5) leaving a total sample of 30 (mean ± standard deviation (SD); Age (years): 20 ± 2; height (cm): 181 ± 7; body mass (kg): 92 ± 17).

### 2.2. Experimental Design

**Figure 1:**
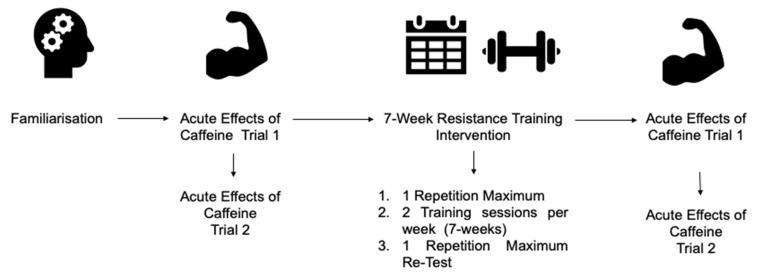
Schematic of experimental design.

Following baseline assessments of maximal strength and a familiarisation trial, the acute effects of caffeine on maximal strength were assessed, which was immediately followed by chronic caffeine consumption through a resistance training program (Figure 1). Finally, the acute effects of caffeine on muscular strength were reassessed following completion of the training program.

Prior to the assessment of the acute effects of caffeine, participants were asked to abstain from caffeine at least 12 h before testing and intense physical activity at least 48 h prior. As outlined below, when prescribed participants consumed caffeine (3 mg·kg^−1^; Myprotein, UK) or a placebo (3 mg·kg^−1^; maltodextrin; Myprotein, UK) issued in a transparent capsule (Bulk^TM^, UK). The ergogenic effect of caffeine supplementation at this dose is well established [3,22,23], and also closely represents a dose achievable without the need for caffeine anhydrous.

### 2.3. Acute Effect of Caffeine on Upper and Lower Body Strength

The acute effects of caffeine on muscular strength were assessed using a double-blind, randomised, and counterbalanced within-subject experimental design. For this part of the study, the participants were asked to visit the human performance laboratory at Coventry University for one familiarisation and two experimental trials (i.e., caffeine and placebo ingestion).

### 2.4. Familiarisation

The first visit was to familiarise participants with the experimental procedures to be used for the assessment of the acute caffeine effect. Initially, assessments were made barefoot in shorts and a t-shirt, and measures of height (cm) and body mass (kg) were taken using a SECA 213 portable stadiometer (SECA 213, Hamburg, Germany) and electronic weighing scales (SECA 803, Hamburg, Germany), respectively. Participants were then asked to complete a caffeine consumption questionnaire [24] to determine typical caffeine consumption habits and a caffeine expectancy questionnaire [25] to determine their perception of caffeine as a performance enhancer. The caffeine expectancy questionnaire consisted of 47 questions, which were categorised into the following: withdrawal/dependence (12 questions), energy/work enhancement (8 questions), appetite suppression (5 questions), social mood enhancement (6 questions), physical performance enhancement (3 questions), anxiety/negative physical effect (9 questions), and sleep disturbance (4 questions). Participants were asked to respond to statements using the following cues: “very unlikely”, “unlikely”, “a little unlikely”,” a little likely”, “likely”, and very likely”. These answers were then transformed into a number with 1 being “very unlikely” and 6 being “very likely” [25]. Scores from each subsection were averaged for each individual.

### 2.5. Warm-Up

Prior to completion of the physical assessments, participants were asked to complete a standardised warm-up of the upper and lower body. The upper body warm-up consisted of 5 min of arm crank ergometry (Lode, Angio) with an unloaded cradle fixed at 70 revs·min^−1^, followed by static and dynamic stretching of the elbow flexors (biceps brachii and brachialis). Then, the participants completed a lower-body warm-up that consisted of 5 min of cycling on an exercise ergometer (Monark 824E Ergomedic) with an unloaded cradle fixed at 70 revs·min^−1^ immediately followed by static and dynamic stretching, focusing on the knee extensors (vastus intermedius, vastus medialis, vastus lateralis, and rectus femoris).

### 2.6. Isokinetic Strength Assessment

After completing the warm-up procedure, participants completed the strength assessment. Skeletal muscle contractile performance was assessed using isokinetic dynamometry in accordance with previously published protocols [18,20,22]. Isokinetic dynamometry is commonly used to evaluate muscular strength [26] and has shown good test-retest reliability in similar populations [27,28]. Furthermore, evidence suggests that a single familiarisation session in athletes adequately addresses potential learning effects for measures of peak torque [29].

Maximal voluntary isokinetic torque (Nm) of the elbow flexors and extensors for the dominant side was measured using an isokinetic dynamometer (Humac Norm, CSMi, model 502140, Stoughton, MA, USA) set up following the manufacturer’s instructions. The rotational axis of the dynamometer head was aligned with the lateral epicondyle of the humerus on the dominant side, with an elbow rest positioned relative to this. A handgrip bar at the opposing end of the lever arm was adjusted relative to the length of the hand and forearm to allow the participant a comfortable grip. During concentric measures, participants were instructed to pull upwards on the bar as hard a possible through a fixed range of motion (80°–120° relative to anatomic zero). During eccentric measures, participants were asked to resist the movement of the lever arm moving through the same range of motion. Average and maximal concentric and eccentric forces were measured at fixed angular velocities of 60, 120, and 180 deg/s. Participants warmed up with 3 submaximal attempts at each speed to become familiarised with the movements and test speeds. During the assessment of maximal voluntary torque, participants performed 3 attempts separated by 60-s rest. The best attempt of the three was used for the analysis. Each speed was separated by a two-minute rest period. Following the measure of maximal concentric and eccentric force, the participants performed 3 sets of 10 maximal repetitions at 60 deg/s with individual repetitions being summed across the 30 repetitions. Each set was separated by 10 s. All torque values collected were corrected for gravity effects by estimation of limb weight prior to the assessment of maximal voluntary torque. Following each set, Rate of Perceived Exertion (RPE) was measured [30].

Participants then completed an assessment of the maximal voluntary isokinetic torque (Nm) of the knee flexors and extensors. Each participant was strapped to the dynamometer chair in a seated position, and the lever arm axis of rotation was aligned with the lateral femoral epicondyle of the dominant limb. The distal end of the lever arm was fitted with a shin pad which was aligned with the lateral malleolus. A strap was placed across the midpoint of the upper limb of the participant’s dominant leg. Throughout the testing, participants were instructed to keep their arms fixed across the chest. The range of motion was fixed at 10°–80° relative to anatomic zero. The testing protocol was then carried out in the way that described for the assessment of maximal voluntary torque of the elbow flexors and extensors.

### 2.7. Experimental Trials

**Figure 2:**
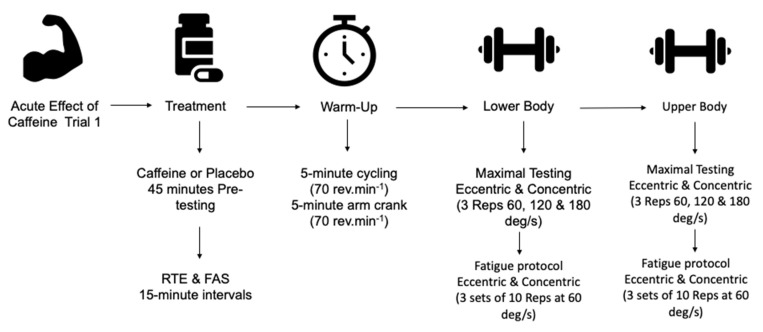
Schematic of the experimental trial.

The experimental protocol followed the procedure outlined above but was proceeded by a treatment ingestion period (Figure 2). Participants consumed either 3 mg·kg^−1^ caffeine or a placebo 45 min prior to completion of the warm-up. Treatments were issued 45 min prior to the warm-up so that the experimental trial commenced 60 min post-ingestion. Previous work indicates that maximal blood plasma concentration of caffeine occurs 60 min post-consumption [23]. Readiness to Invest Effort (RTE) [31] and Felt Arousal Scale (FAS) [32] were measured pre-consumption, 15, 30, and 45 min after consumption. An analogue scale was used to measure RTE with a scale from “not ready at all” to “totally ready”. A scale of 1–6 was used for FAS with 1 being “low arousal” and 6 being “high arousal”.

### 2.8. Chronic Effects of Caffeine Ingestion during a 7-Week Resistance Training Program

**Figure 3:**
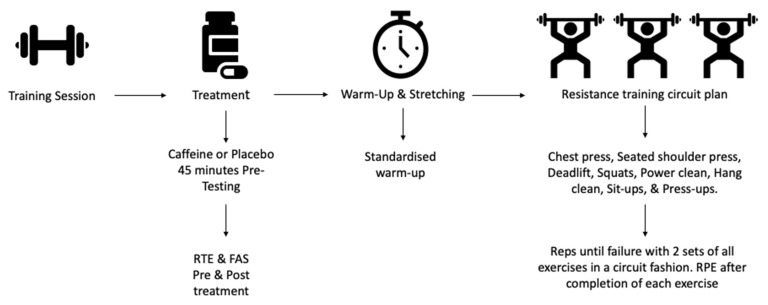
Schematic of Resistance training sessions.

To assess if chronic caffeine ingestion can augment adaptations to resistance training, a between subject’s experimental design was conducted. Participants were randomly split into a control (*n* = 15) or caffeine (*n* = 15) group. Groups were matched based on habitual caffeine use, using the data obtained from the caffeine consumption questionnaire, and for baseline strength, using the procedures outlined in the statistical method. Following the assessment of 1RM, all participants then completed the same training regime as indicated below (Figure 3).

### 2.9. Assessment of 1 Repetition Maximum

1RM values in the six exercises were assessed over two visits to program the training regime and as an additional baseline marker of muscular strength. On the initial visit, the researcher provided a demonstration of the correct lifting techniques for all used exercises. For all exercises, participants then completed 8–10 unweighted repetitions to ensure the correct lifting technique was achieved. Participants completed assessments of 1RM for squats (SQ), deadlifts (DL), chest press (CP), seated shoulder press (SSP), power clean (PC), and hang clean (HC). All exercises were completed using a 20 kg Eleiko barbell and in accordance with published protocols [33,34,35]. Prior to 1RM attempts participants started at 50% of estimated 1RM for 3–5 repetitions, progressing to 70% for 1–3 repetitions, and 90% for 1 repetition. All 1RM values were determined by progressively increasing the weight (e.g., 5 kg per attempt) lifted until the participant failed to lift the set weight through a full range of motion and using the correct form [36,37]. A trained researcher/ spotter was present during all testing sessions to ensure a proper range of motion. Any lift that deviated from proper technique was not counted. This included a lack of full range of motion exhibited during the lift or technique that did not conform to guidelines for the execution of the exercise in question as defined by Baechle and Earle [38]. This procedure was then repeated on the second visit, which occurred at least 2 days later. A minimum of 1 min of rest was permitted between attempts and a minimum of 5 min rest between lifts. Lifts were altered between the upper and lower body to reduce fatigue and their order was consistent in each testing session.

### 2.10. Resistance Training Intervention (7-Weeks)

The effect of caffeine on resistance training was assessed using a double-blind, between-subject experimental design. Participants were asked to abstain from caffeine 12 h prior to the commencement of each training session. Forty-five minutes before the commencement of each training session participants in the caffeine group consumed 3 mg·kg^−1^ caffeine and those in the placebo group an equivalent dose of maltodextrin in the same manner as previously outlined. The intention of the resistance training intervention was to develop upper and lower body maximal strength and strength endurance. Following a standardised warm-up consisting of static and dynamic stretches focusing on the upper and lower body muscles participant completed a circuit training program consisting of 8 exercises. The exercises were CP, SSP, SQ, DL, PC, HC, sit up, and press-ups. The load for CP, SSP, SQ, DL, PC, HC was set at 70% of 1RM; sit-ups and press-ups were performed without any external load. Throughout the course of the intervention, all exercises were performed with “repetitions until failure” (RTF), meaning that the participants lifted 70% of 1RM until exhaustion. Previous resistance training interventions commonly used loads of 60–80% of 1RM [39,40,41,42] given that such loads have been shown to increase muscular strength [38,43]. Previous work has demonstrated that resistance training intervention implementing repetitions until failure protocols are effective for improving muscular strength and strength endurance [44,45,46]. Repetitions until failure was favoured over protocols using a fixed training load given that the open-ended nature of repetitions until failure protocol allowed for a more robust assessment of the effect of acute caffeine consumption across the course of the training intervention. All participants completed two circuits of all 8 exercises, alternating between upper and lower body exercises. A minimum of 2 min rest was provided between exercises and 10 min rest between circuits. The number of successful repetitions and RPE were collected after each exercise. Circuit training sessions took place Tuesday morning and Thursday evening for 7-weeks. Previous work has shown that resistance-training interventions of similar duration are effective in increasing muscular strength [9,41,47]. Upon completion of the 7-week intervention training programme, all participants completed reassessments 1RM.

### 2.11. Statistical Analysis

Data analysis was performed using Statistical Package for the Social Sciences (IBM SPSS Statistics Version 25) and Excel (Microsoft Windows Version 16.41 2020). Initially, appropriate tests of normality and homogeneity were performed. To match groups for baseline strength prior to completion of the resistance training program, a Pearson’s or Spearman’s correlation was performed between all measures of 1RM. All measures, other than DL and PC (R = 0.364 *p* = 0.048) and DL and HC (R = 0.239, *p* = 0.203), were significantly correlated (R = 0.463–0.820, *p* < 0.05 in all cases). Given that 28 of 30 1RM comparisons demonstrated a significant correlation, all the data were transformed to a Z-score using the formula (x-μ)/σ, summed and compared using an independent sample t-test. The acute effects of caffeine on measures of peak torque (PT), total work (TW), and RPE, were assessed using a 4-factor mixed-model ANOVA. The between factor being Group (caffeine and placebo) and within factors being Time (pre-and post-intervention), Treatment (caffeine or placebo), and Speed/Set (60, 120, and 180/Set 1, Set 2, and Set 3). Furthermore, RTE and FAS were assessed using a 4-factor mixed-model ANOVA. The between factor being Group (caffeine and placebo) and within factors being Time (pre-and post-intervention), Treatment (caffeine or placebo), and Minutes (pre, 15, 30, and 45-min post-ingestion). Maximal strength (i.e., 1RM) measures were assessed using a single factor ANOVA with the fixed factor of Time (pre-and post-intervention). All intervention data (RTF and RPE) were analysed using an imputation method [48,49] followed by a 3-factor mixed model ANOVA with a between factor of Group (caffeine or placebo) and within factors of Session (1–14) and Set (Set1 and Set2). All violations of sphericity were adjusted using Greenhouse–Geisser where appropriate. Relevant main effects and significant interactions were further analysed in a Bonferroni adjusted pairwise comparison. Cases of violated normality were present; however, ANOVA was still considered a robust method of statistical analysis in such cases [50]. Partial eta squared (η^2^) was reported with significant ANOVA main effects as a measure of effect size [51]. Additionally, effect size (*d*) from the pairwise comparison (bias-corrected (Hedges) was calculated using the difference in means divided by the SD of the compared variables. Effect size was reported using the following categories: trivial < 0.20, small 0.20–0.49, medium 0.50–0.79, and large > 0.80 [52]. Data were presented as Mean ± SD with statistical significance set at a level of *p* < 0.05.

## 3. Results

### 3.1. Participation Characteristics

Age (caffeine group: 20 ± 2 years; placebo group: 19 ± 2 years; *p* = 0.66), height (caffeine group: 181 ± 8 cm; placebo group: 181 ± 5 cm; *p* = 0.78), or body mass (caffeine group: 89 ± 10 kg; placebo group: 95 ± 22 kg; *p* = 0.30) did not differ between individuals assigned to the caffeine or placebo groups. Average caffeine consumption was 133 ± 123 mg per day and 121 ± 95 mg per day for the caffeine and placebo groups, respectively, with four participants reporting no caffeine use (caffeine group *n* = 2; placebo group *n* = 2). Average caffeine consumption did not differ between groups (*p* = 0.888, *d* = 0.11). The perceived effect of caffeine in energy/work enhancement and sleep disturbance were significantly higher in the placebo group (Table 1: *p* < 0.045, *d* = 0.97); however, there were no other significant differences for any other caffeine expectancy subscale (Table 1: *p* < 0.744, *d* < 0.97). Baseline strength was comparable between the groups prior to completing the resistance training program (*p* = 0.40, *d >* 0.31).

### 3.2. Peak Torque

#### 3.2.1. Elbow Extension Peak Torque

For eccentric PT of the elbow extensors, there was a significant Treatment*Speed interaction (Table 2: *p* = 0.001, η_p_^2^ = 0.336). Pairwise comparison indicated that eccentric PT at 120 and 180°/s was higher following caffeine supplementation compared to placebo (Table 2: *p* < 0.013, *d >* 0.24). Furthermore, eccentric PT was greater at 180°/s compared to that at 60 and 120°/s, but only in the caffeine trial (Table 2: *p* < 0.005, *d* > 0.17). For both concentric and eccentric PT there were no other significant interactions (Table 2: *p* > 0.055, η_p_^2^ < 0.098). Concentric PT was significantly affected by Speed (Table 2: *p* = 0.001, η_p_^2^ = 0.708) with PT at 180°/s being lower than that at 60 and 120°/s (Table 2: *p* < 0.001 in both cases). There were no other significant main effects (Table 2: *p* > 0.208, η_p_^2^ < 0.088).

#### 3.2.2. Elbow Flexion Peak Torque

For concentric PT of the elbow flexors, there was a significant Group*Time*Treatment interaction (Table 2: *p* = 0.024 η_p_^2^ = 0.168) and for both concentric and eccentric PT a significant Time*Treatment*Speed interactions (Table 2: *p* < 0.034 η_p_^2^ > 0.112). Pairwise comparisons indicated that for the caffeine group, concentric PT was higher post intervention and increased in the placebo group but only when the pre to post caffeine trail was compared (Table 2: *p* < 0.008, *d* > 0.17). Eccentric PT was not significantly increased post-intervention (Table 2: *p* > 0.094, *d* < 0.53) and was not affected by acute caffeine treatment either pre- or post-the intervention (Table 2: *p* > 0.209, *d* < 0.35). However, acute caffeine treatment increased concentric PT measured at 60 and 120°/s both pre and post the training intervention (Table 2: *p* < 0.005, *d* > 0.19). In some cases, PT was affected by speed. Following the intervention concentric PT at 180°/s was higher than that at 60 and 120°/s (Table 2: *p* < 0.003 in both cases) and following the placebo treatment PT at 60°/s was higher than 120 and 180°/s (Table 2: *p* < 0.030, *d* > 0.09).

#### 3.2.3. Knee Extension Peak Torque

For eccentric PT of the knee extensors, there was a significant Group*Time*Treatment*Speed interaction (Table 2: *p* = 0.016 η_p_^2^ = 0.138). Pairwise comparison indicated that the caffeine group had an increase in PT at 60°/s post the exercise intervention at a level that was approaching significant (Table 2: *p* = 0.053, *d* > 0.22), whereas the placebo group had no significant training effect (Table 2: *p* = 0.083, *d* > 0.09). There was no effect of acute caffeine supplementation or speed (Table 2: *p* > 0.166 η_p_^2^ < 0.001). For concentric PT, there was a significant Group*Time interaction (Table 2: *p* = 0.010 η_p_^2^ = 0.212). Pairwise comparison indicated PT was improved following the intervention in the caffeine group (Table 2: *p* = 0.010, *d* > 0.27) but unchanged in the placebo group (Table 2: *p* = 0.275, *d* < 0.47). For concentric PT, there were no other significant interactions (Table 2: *p* > 0.097 η_p_^2^ < 0.114) or a main effect of acute caffeine treatment (Table 2: *p* = 0.344 η_p_^2^ = 0.114). However, PT was significantly affected by Speed (Table 2: *p* = 0.001 η_p_^2^ = 0.665) with PT at 180°/s being lower than that at 60 and 120°/s (Table 2: *p* < 0.001 in both cases *d* > 0.27).

#### 3.2.4. Knee Flexion Peak Torque

For concentric and eccentric PT of the knee flexors there were no significant interactions (Table 2: *p* > 0.094 η_p_^2^ > 0.002) and no main effect of Group (Table 2: *p* > 0.417 η_p_^2^ > 0.004). For concentric PT, there was no main effect of Treatment (Table 2: *p* = 0.075 η_p_^2^ = 0.109), but there was a main effect of Time (Table 2: *p* = 0.007 η_p_^2^ = 0.230) with PT post the exercise intervention being significantly higher than pre-intervention exercise. There was also a main effect of Speed (Table 2: *p* = 0.001 η_p_^2^ = 0.418) with performance at 180°/s being lower than that at 60 and 120°/s (Table 2: *p* < 0.003 in both cases *d* > 0.16). For eccentric PT, there was no main effect of Speed (Table 2: *p* = 0.316 η_p_^2^ = 0.040). However, there was a main effect with treatment (Table 2: *p* = 0.007 η_p_^2^ = 0.223) where acute caffeine increased eccentric PT compared to the placebo trial.

### 3.3. Total Work

#### 3.3.1. Elbow Extension Total Work

For concentric and eccentric TW of the elbow extensors there was no significant interactions (Table 3: *p* > 0.065 η_p_^2^ > 0.003), no main effects of Group (Table 3: *p* > 0.762 η_p_^2^ > 0.003), Treatment (Table 3: *p* > 0.151 η_p_^2^ > 0.072), or Time (Table 3: *p* = 0.578 η_p_^2^ > 0.006). However, there were significant main effects of Set (Table 3: *p* = 0.001 η_p_^2^ > 0.527) with performance at Set1 being higher than that at Set2 and Set3 (J) (Table 3: *p* < 0.001, *d* > 0.36) and performance at Set2 being higher than that at Set3 (Table 3: *p* = 0.001, *d* > 0.33).

#### 3.3.2. Elbow Flexion Total Work

For concentric and eccentric TW of the elbow flexors, there was a significant interaction between Time*Set (Table 3: *p* < 0.047 η_p_^2^ > 0.113). Pairwise comparison for concentric TW indicated that pre-exercise intervention, Set1 TW was higher than post-intervention (Table 3: *p* = 0.002, *d* > 0.30). Furthermore, both pre-and post-the intervention performance in Set2 and Set3 was significantly lower than at Set1 (Table 3: *p* < 0.001) for both concentric and eccentric TW. No other significant interactions were identified (Table 3: *p* > 0.096 η_p_^2^ < 0.931) and there was no main effect of Group (Table 3: *p* > 0.271 η_p_^2^ < 0.035). Concentric TW was not affected by Treatment (Table 3: *p* = 0.231 η_p_^2^ = 0.051); however, there was a main effect of Treatment for eccentric TW (Table 3: *p* = 0.003 η_p_^2^ = 0.272), where TW following acute caffeine treatment was greater than the placebo treatment.

#### 3.3.3. Knee Extension Total Work

For eccentric TW of the knee extensors there were no significant interactions (Table 3: *p* > 0.130 η_p_^2^ > 0.032). For concentric TW there was a significant Time*Treatment interaction (Table 3: *p* = 0.001 η_p_^2^ = 0.350). Pairwise comparison indicated an increase in TW post-intervention (Table 3: *p* = 0.001, *d* < 1.04). Furthermore, TW was significantly increased in the caffeine trial prior to the exercise intervention (Table 3: *p* = 0.001, *d* < 1.34), however, post-intervention there was no significant difference between the caffeine and placebo trial (Table 3: *p* = 0.599, *d* < 0.18). For concentric TW there were no other significant interactions (Table 3: *p* > 0.184 η_p_^2^ < 0.933). For both concentric and eccentric TW there was no effect of Group (Table 3: *p* > 0.494 η_p_^2^ < 0.018). Eccentric TW reported no other main effects (Table 3: *p* > 0.140 η_p_^2^ < 0.077) but for concentric TW there was a main effect of Set (Table 3: *p* = 0.001 η_p_^2^ = 0.400). TW at Set1 was higher than Set2 and Set3 (Table 3: *p* < 0.010, *d* > 0.11) and performance at Set2 was higher than that at Set3 (Table 3: *p* = 0.001, *d* > 0.009).

#### 3.3.4. Knee Flexion Total Work

For concentric TW of the knee flexors there were no significant interactions (Table 3: *p* > 0.113 η_p_^2^ < 0.075), but for eccentric TW there was a Group*Treatment*Set interaction (Table 3: *p* = 0.029 η_p_^2^ = 0.119). Pairwise comparison indicated that there was no effect of Group (Table 3: *p* > 367, *d* = 0.12–2.00). However, eccentric TW was greater in the placebo group during caffeine trials (Table 3: *p* = 0.046, *d* > 0.13). In the caffeine group Set1 TW was significantly higher during the caffeine trials (Table 3: *p* = 0.029, *d* > 0.26). For eccentric TW there was no main effect of Time (Table 3: *p* = 0.007 η_p_^2^ = 0.235) and for concentric TW there was no main effect of Group (Table 3: *p* = 0.256 η_p_^2^ = 0.046) or Time (Table 3: *p* = 0.148 η_p_^2^ = 0.073). However, concentric TW was significantly increased following caffeine treatment (Table 3: *p* = 0.014 η_p_^2^ = 0.198). Concentric and eccentric TW was effected by Set (Table 3: *p* = 0.001 η_p_^2^ = 0.629) with performance at Set1 being higher than that at Set2 and Set3 (Table 3: *p* < 0.010, *d* > 0.61) and performance at Set2 being higher than that at Set3 (Table 3: *p* = 0.001, *d* > 0.014).

### 3.4. Rate of Perceived Exertion

#### 3.4.1. Elbow Extension Rate of Perceived Exertion

RPE following repeated MVC of the elbow extensors there were significant Group*Time (Table 4: *p* = 0.025 η_p_^2^ = 0.167), Time*Treatment (Table 4: *p* = 0.003 η_p_^2^ = 0.267), Time*Set (Table 4: *p* = 0.011 *p* = 0.025 η_p_^2^ = 0.186), and Treatment*Set (Table 4: *p* = 0.050 *p* = 0.025 η_p_^2^ = 0.115) interactions. Pairwise comparison indicated no group difference in RPE following MVC (Table 4: *p* < 0.437, *d* < 0.62). The caffeine group had increased RPE following MVC post-training intervention (Table 4: *p* = 0.001 *d* > 0.097). Furthermore, the placebo group had a treatment effect post-intervention, with RPE during the caffeine trial being higher than the placebo trial (Table 4: *p* < 0.006) at Set2 (Table 4: *d* = 0.87) and Set3 (Table 4: *d* = 0.76). Irrespective of treatment, RPE following Set3 was higher than that following completion of Set1 and Set2 (Table 4: *p* = 0.001 *d* > 0.97.) There were no other significant interactions (Table 4: *p* > 0.085 η_p_^2^ < 0.936).

#### 3.4.2. Elbow Flexion Rate of Perceived Exertion

For RPE following repeated MVC of the elbow flexors, there was a significant Time*Set interaction (Table 4: *p* = 0.001 η_p_^2^ = 0.222). Pairwise comparisons indicated an increase in RPE following MVC post-exercise intervention (Table 4: *p* = 0.001 *d* > 0.48). Both pre-and post-exercise intervention, RPE following Set 3 was higher than that following Set 1 and Set 2 (Table 4: *p* < 0.001 *d* > 0.62). There were no other significant interactions (Table 4: *p* > 0.072 η_p_^2^ < 0.064) and no main effect of Group (Table 4: *p* = 0.771 η_p_^2^ < 0.014) or Treatment (Table 4: *p* = 0.961 η_p_^2^ = 0.001).

#### 3.4.3. Knee Extension Rate of Perceived Exertion

For RPE following repeated MVC of the knee extensors, there was a significant Group*Time*Set interaction (Table 4: *p* = 0.017 η_p_^2^ = 0.135). Pairwise comparison indicated no group effect pre-or post-exercise intervention (Table 4: *p* > 0.130 *d* < 2.18). However, both treatment groups indicated increased RPE post the exercise intervention across (Table 4: *p* < 0.001 *d* > 0.40). RPE following Set3 was significantly higher than Set1 and Set2 (Table 4: *p* < 0.001 *d*< 2.18.). There was also a Time*Treatment interaction (Table 4: *p* = 0.001 η_p_^2^ = 0.396), where RPE was higher post-intervention in both treatments (Table 4: *p* = 0.001 *d* > 0.97). Furthermore, prior to the exercise intervention, RPE was significantly higher following caffeine treatment (Table 4: *p* = 0.001 *d* > 0.76).

#### 3.4.4. Knee Flexion Rate of Perceived Exertion

For RPE following repeated MVC of the knee flexors, there was no significant interaction (Table 4: *p* > 0.061 η_p_^2^ < 0.975). However, there was a main effect with Time (Table 4: *p* = 0.001 η_p_^2^ = 0.748) with post-training intervention RPE being increased. There was also a main effect with Set (Table 4: *p* = 0.001 η_p_^2^ = 0.898) with RPE following Set3 being higher than Set1 and Set2. There were no main effects of Group (Table 4: *p* = 0.760 η_p_^2^ = 0.002) or Treatment (Table 4: *p* = 0.666 η_p_^2^ = 0.006).

### 3.5. Readiness to Invest Effort and Felt Arousal Scale

For RTE Physical, RTE Mental, and FAS there were no significant interactions (Table S1: *p* > 0.060 η_p_^2^ > 0.001). RTE Physical, RTE Mental, and FAS were significantly affected by time post-ingestion (Table S1: *p* < 0.001 η_p_^2^ > 0.245). Both RTE Physical and FAS reported 45 min post-treatment were significantly higher than pre, 15- and 30-min post-treatment (Table S1: *p* < 0.015 in all cases *d* < 0.68). However, RTE Mental 30 min post-treatment was higher than pre, 15-, and 45-min post-treatment (Table S1: *p* < 0.050 in all cases *d* < 0.30). There were no significant main effects of Group (Table S1: *p* > 0.150 η_p_^2^ > 0.007), Time (Table S1: *p* > 0.057 η_p_^2^ > 0.124) or Treatment (Table S1: *p > 0*.415 η_p_^2^ > 0.003).

### 3.6. 1RM

**Figure 4:**
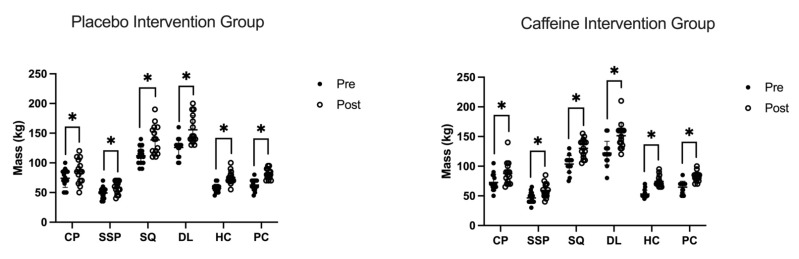
Effect of caffeine treatment (3 mg·kg^−1^) pre and post-7-week resistance training program on pre-intervention 1RM and post-intervention 1RM, * represents a significant increase in performance post exercise intervention. The post intervention increase in 1RM was 16% and 16% CP; 19% and 22% SSP; 24% and 25% SQ; 24% and 24% DL; 42% and 40% HC; 30% and 27% PC for the placebo intervention group and caffeine intervention group, respectively. There was no main effect of intervention group for each exercise (*p* > 0.850) other than the DL (*p* = 0.001).

For 1RM performance, there were no significant Group*Time interaction (Figure 4: *p* < 0.962, η_p_^2^ < 0.008). Following the resistance training intervention, 1RM improved in all lifts (Figure 4: *p* < 0.001 η_p_^2^ > 0.633). There was no significant effect of Group in all exercises (Figure 4: *p* > 0.850 η_p_^2^ < 0.071) apart from DL (Figure 4: *p* = 0.001 η_p_^2^ = 0.493) with the placebo group having a greater performance during 1RM assessment.

### 3.7. Intervention Repetitions until Failure

**Figure 5:**
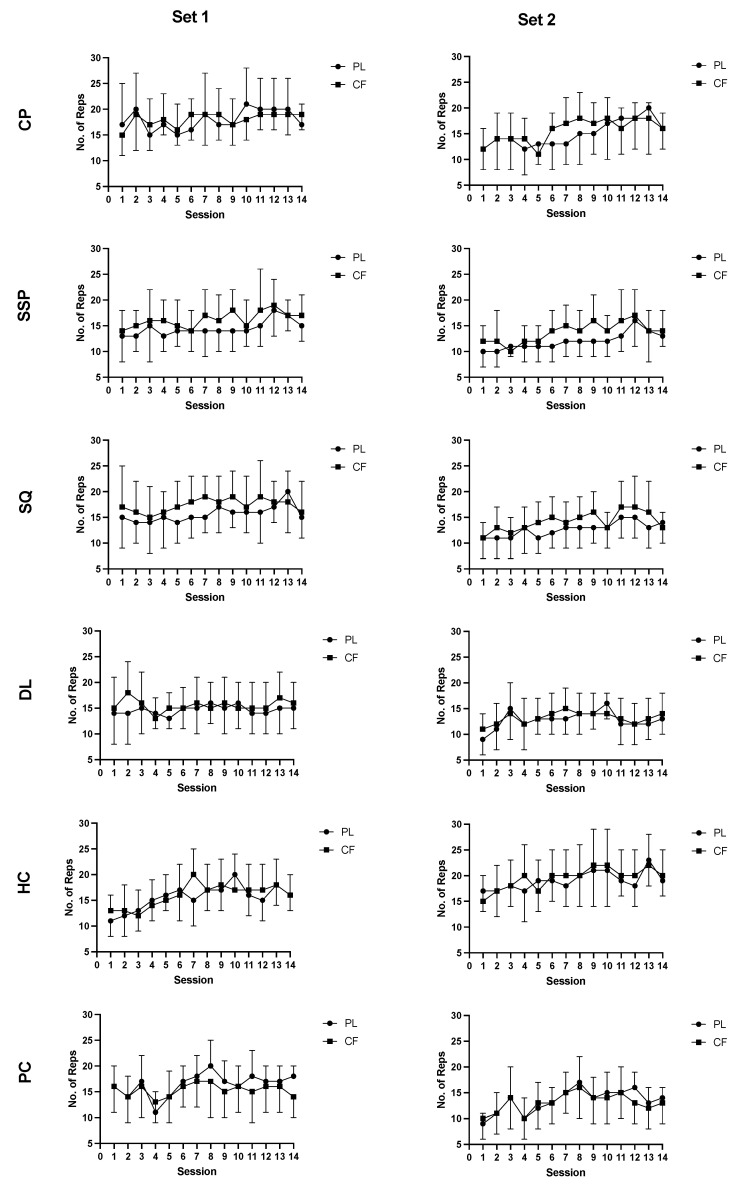
Effect of caffeine treatment (3 mg·kg^−1^) during the 7-week resistance training program on sessional RTF.

For all exercises, there were no significant interactions (Figure 5: *p* = 0.998). However, RTF across the training intervention was higher in the caffeine group for SSP, SQ, DL, and HC (Figure 5: *p* < 0.037 *d* < 0.13). The placebo group outperformed the caffeine group in PC (Figure 5: *p* = 0.005 *d* < 0.18), and there were no between-group differences for CP (Figure 5: *p* = 0.618). For all exercises, there was also a main effect of Session (Figure 5: *p* < 0.005). Whilst there were some differences between specific sessions, there were clearer trends for an increase in CP, SSP, DL, and PC RTF as the duration of the intervention increased. Furthermore, for all exercises, there was a main effect of Set with RTF being higher in Set1 compared to Set2 (Figure 5: *p* < 0.001 *d* < 0.14).

### 3.8. Intervention RPE

For both SQ and HC, sessional RPE of the caffeine group was lower than that of the placebo group (Table S2: *p* < 0.035 *d* < 0.62). For CP, SQ, and DL there was a main effect of Session (Table S2: *p* < 0.034). Pairwise comparisons show some session-specific differences for SQ and DL, but no clear trends. Furthermore, for all exercises, there was a main effect of Set, with RPE being higher in Set1 compared to Set2 (Table S2: *p* < 0.044 *d* > 0.49). There were no significant interactions (Table S2: *p* > 0.072).

## 4. Discussion

Data from the present study indicate that prior to the training intervention the acute ingestion of 3 mg·kg^−1^ caffeine increased specific measures of muscular strength in a muscle- and contractile mode-specific manner. Twice weekly ingestion of caffeine during the 7-week resistance-training program had little effect on caffeine’s ability to evoke an acute benefit, with many of the reported caffeine-induced improvements in muscular strength still prevalent in both the caffeine and placebo groups when reassessed upon completion of the training intervention. Although the seven-week resistance training intervention significantly improved 1RM and specific measures of maximal isokinetic torque—other than the PT of the knee extensors, which was only improved in the caffeine treated group—benefits were not statistically different between the group that consumed caffeine prior to each session compared to those that consumed a placebo. This similar increase in performance upon completion of the exercise intervention was apparent despite the greater number of resistance exercise repetitions performed for numerous exercises in the caffeine group across the course of the intervention. As such, the results of this study demonstrate that: (1) 3 mg·kg^−1^ may be useful to evoke acute enhancements in muscular strength; (2) relatively short-term chronic ingestion of 3 mg·kg^−1^ of caffeine may not lead to habituation to its effects; (3) chronic caffeine supplementation for 7-week may not enhance resistance-training induced gains in strength.

### 4.1. Acute Effects of Caffeine on Muscular Strength

Recent meta-analyses indicate that acute caffeine consumption may enhance muscular strength, including strength assessed using isokinetic dynamometry [1,3,5]. In line with these findings, the results of the present study indicate that acute caffeine ingestion increased specific measures of PT and TW when compared to a placebo. More specifically, an acute ergogenic effect of caffeine was observed for: (a) eccentric PT of the elbow extensors at higher angular velocities and concentric PT of the elbow flexors; (b) elbow flexion TW; (c) eccentric PT of the knee flexors; and (d) knee extension TW (pre-intervention) and knee flexion TW (both pre-and post-intervention). Based on these data and in accordance with previous work [17,18,19,20], it can be concluded that caffeine has a muscle- and contractile mode-specific effect. Whilst our data indicate caffeine-induced benefits for both concentric and eccentric PT, there appears to be a greater number of benefits for upper body musculature. This contradicts previously published work indicating superior benefits for lower limb musculature, attributed to mechanisms of the central nervous system stemming a greater increase in muscular recruitment within larger muscle groups [7]. Conversely, a more recent meta-analysis by Grgic et al. [3] indicated that caffeine effects on strength and power were more evident in the upper body.

Mechanistically, the ability of caffeine to evoke improved exercise performance is primarily attributed to its effects as a central adenosine receptor antagonist [53,54,55,56]. Caffeine and adenosine share similar molecule structures. Because of their similar structure, they compete for adenosine binding sites resulting in a surge excitatory neurotransmitter causing reduced pain perception [22,57], a rise in motor unit recruitment, and improved excitation-contraction coupling [22,58,59]. Caffeine may act directly on skeletal muscle [60,61,62,63], increasing the release and myofibril sensitivity to Ca^+2^ [64,65,66], resulting in increased cross-bridge formation. In support, the exposure of isolated skeletal muscle to physiological concentrations of caffeine has been shown to evoke increased muscular power [22].

Although it was not the purpose of the present study to determine the mechanisms underpinning the acute effects of caffeine on muscular strength, the influences of some of these mechanisms can be determined. When measured in the arm of the experiment examining the acute effects of caffeine, post-exercise RPE was in the most part unaffected by caffeine, and on some occasions was increased following acute caffeine ingestion. This coincides with previous work where caffeine ingestion had a limited effect on perceived effort or pain in scenarios where exercise performance was unchanged [39,67,68] or improved [69,70,71] following caffeine consumption. Intersessional RPE following CP and PC exercise was not different between the groups. However, RPE following SSP, SQ, DL, and HC was reduced in the caffeine group. This may in part support the concept of caffeine as a pain suppressor in some circumstances, though this should be interpreted with caution given that this part of the protocol used a between subject’s design.

Interestingly, caffeine did not have an effect on RTE physical and mental effort when measured in the arm of the experiment examining the acute effects of caffeine. This coincides with previous research [68,72]. Whilst these findings point to physiological mechanisms being the cause of the documented effects, the impact of caffeine on arousal during the exercise as a mechanism cannot be ruled out. To some extent, the results of perceptual measures needed to be interpreted with caution given their sensitivity to change following caffeine ingestion have not been robustly explored.

### 4.2. Habituation to Chronic Caffeine Consumption

There is a debate in the literature as to whether the acute benefits of caffeine on physical performance are reduced following chronic exposure, with some evidence indicating habituation to caffeine’s effect [12,13] and others suggesting no habituation effects [11,73,74,75]. In the present study, the acute effects of caffeine on PT and TW were reasonably uniform prior to and following the training intervention. For example, acute caffeine-induced increases in eccentric elbow extension PT, concentric elbow flexion PT, eccentric knee flexion PT, eccentric elbow flexion TW, and concentric knee flexion TW that was demonstrated prior to the intervention were still prevalent in the caffeine intervention group when acute caffeine responses were reassessed post-intervention.

These results suggested that consuming 3 mg·kg^−1^ of caffeine, twice per week for 7-weeks, in a population of 30 moderate habitual caffeine consumers, did not reduce the caffeine’s ergogenicity. Although there are varying methodological approaches to assess caffeine habituation, the findings of the present work agree with previous evidence that indicates that caffeine evokes equivalent performance-enhancing effects in low and high habitual caffeine users [1,8,11,74,76]. For example, Gonçalves et al. [11] demonstrated that 6 mg·kg^−1^ of caffeine improved cycling time trial performance in low (58 mg/day), moderate (143 mg/day), and high (351 mg/day) habitual caffeine users. Still, although models stratifying participants by typical caffeine consumption habits for examining the potential for habituation have value, the results from these studies may be limited by the reliance on subjective caffeine recall used to quantify caffeine use.

The disparity between the current data and studies that have directly measured and have indicated habituation to caffeine effects is likely due to differences in the population characteristics, the dose, and duration of caffeine administration, and the outcome variables assessed. For example, Beaumont et al. [13] included low habitual caffeine consumers (<75 mg/day) randomly assigned to consume placebo or caffeine (1.5–3 mg·kg^−1^) for 28 days. Results indicated that the acute effect of caffeine on cycling performance was evident pre-supplementation and was no longer apparent after 4 weeks of caffeine supplementation. Lara et al. [12] used a caffeine dose of 3 mg·kg^−1^ supplementation for 20 consecutive days, during which performance in a Wingate test was evaluated. While caffeine increased cycling power, the ergogenic effects attenuated over the course of the study, suggesting progressive tolerance. Indirect assessments of the impact of habituation have also be made by studies that stratify their participant groups by habitual caffeine use. Several studies demonstrate an ergogenic effect in high caffeine users across anaerobic and aerobic modes of exercise [1,11,73,74,76]. Studies examining caffeine habituation specifically for measures of muscular strength are limited, and as such, these findings make a novel contribution to the literature.

### 4.3. Effects of Chronic Caffeine Consumption on Resistance Training

Irrespective of the supplement provided, the training intervention resulted in a significant increase in all assessments of 1RM and specific measures of PT and TW. The more pronounced effects of the training intervention of 1RM were expected due to training specificity [77]. Post-intervention concentric PT of the knee extensors at all angular velocities and eccentric PT at 60 deg/s was improved only in the caffeine group. Other improvements in PT, TW, and measures of 1RM were uniform across the caffeine and placebo-treated groups, indicating a limited effect of chronic caffeine ingestion to augment adaptations to resistance exercise. The chronic effect of caffeine as a nutritional strategy to support training has received little attention. Our findings differ from the findings presented by Kemp et al. [9] who demonstrated that strength-trained participants that consumed 3 mg·kg^−1^ caffeine before each session of a 6-week resistance training intervention had superior improvements in the bench press and squat 1RM compared to those that consumed a placebo for the duration of the intervention. However, our data support work by Giraldez-Costas et al. and Pakulak et al. [16,78] who indicated no significant differences in 1RM strength gains between caffeine and placebo groups following 4 or 6 weeks of resistance training intervention.

We progress previous findings by demonstrating this relationship following a longer intervention period and across a larger range of strength assessments considering not only 1RM in different exercises but also maximal voluntary concentric and eccentric muscle functions across a range of joint angular velocities. The disparity between the present findings and those of Kemp et al. [9] may be related to differences between populations. Kemp et al. [9] used specifically strength-trained participants, and it has been suggested that caffeine may have a greater effect on this population as compared to untrained individuals [3,79,80]. Whereas this may in part be related to improved repeatability of performance in specifically trained individuals [3,79,80], this suggestion has some mechanistic underpinning. Previous research suggests that trained individuals have a greater adenosine receptor density compared to those who are less trained or untrained [81]. Those with an increased adenosine receptor density may evoke amplified caffeine [82]. However, this is speculative and requires further investigation in light of evidence reporting similar caffeine-induced benefits in performance in both trained and untrained participants [80,83], and in some cases, increased responses in untrained individuals [80].

This study is the first to monitor the acute effect of caffeine during each session of a training intervention. Interestingly, across the training intervention, RTF was higher in SSP, SQ, DL, and HC for the caffeine group. While this may add further weight to the acute benefits of caffeine, it demonstrates the small but significant increase in the relative total weight lifted was not substantial enough in magnitude to evoke a superior training response. Furthermore, sessional RPE was either unchanged or in some cases decreased in the caffeine group, this again indicates that the ability of caffeine to suppress perception of pain/ effort may be more prevalent during sustained contractions [40,67,68].

## 5. Limitations and Future Direction

The present study offers valuable insight with respect to the effects of chronic caffeine supplementation as a nutrition strategy to augment resistance training, though it is not without limitation. Firstly, a resistance-training program with a weekly training frequency greater than that adopted in the present study would likely evoke greater adaptations in the measured outcomes [84]. However, the training frequency used in the present study was designed to coincide with other commitments of the club. That said, the training regime adopted successfully improved measures of muscular strength and therefore was appropriate to address the experimental aims.

Our data indicate a small but significant increase in training volume across the course of the intervention, whilst this did not manifest in an enhanced training response in the measured outcomes there is the possibility that a sustained increase in training volume over a longer period may evoke enhanced adaptations. This is an avenue for future investigation.

We did not measure genetic influencers that potentially may influence the caffeine’s performance-enhancing effect [3,85,86]. A recent review has indicated that the CYP1A2 and the ADORA2A may be important influencers of the caffeine response [87]. Future work should consider genetic influences that underpin caffeine’s effect when assessing the impact of chronic ingestion as a method to elicit superior training adaptations.

Furthermore, this study used individuals that were physically conditioned but not specifically resistance trained. Although the demonstrated effects may be relevant to a broad population that is relatively naive to resistance training, they may not be generalisable to individuals that are specifically resistance trained. Future work should look to directly compare trained against untrained participants using a similar approach to that adopted in the present study. Future work should also look to repeat this study using different doses of caffeine. We selected 3 mg·kg^−1^ for the present study as it typically considered the minimum dose needed to evoke an improved exercise performance [88,89,90] and more closely represents caffeine consumed from commercially available products. Previous work exploring the acute effects of caffeine on physical performance is focused on males [1,2,3,4,5,7] and there is recent growing interest in establishing the caffeine response in females [91]. Whilst it seems that the effects of caffeine on resistance exercise performance are similar between males and females [92], future similar studies performed with females as study participants are nevertheless needed.

## 6. Conclusions

This study examined the acute effects of 3 mg·kg^−1^ caffeine consumption on muscular strength and explored if chronic use of caffeine could be used as an effective nutrition strategy to augment the response to resistance training. The data presented herein indicate a muscle and contractile mode-specific acute benefit of caffeine consumption, effects that were not dampened following twice-weekly consumption over the time course of a seven-week resistance training intervention. Whilst the training intervention resulted in significant improvements in 1RM performance and specific measures of isokinetic PT and TW of the upper and lower limb musculature, for the most part, the effects were equivalent in both the group ingesting caffeine and in the group ingesting a placebo. These effects were prevalent despite a small but significant increase in RTF across the training intervention in the caffeine group. In summary, our results infer that caffeine may be beneficial to evoke acute improvements in muscular strength but has limited benefits across the course of a resistance training intervention.

## Figures and Tables

**Table 1:** Caffeine expectancy between caffeine and placebo groups.

	Caffeine Intervention Group	Placebo Intervention Group
Caffeine Intake	133 ± 123 mg/day	121 ± 95 mg/day
Withdrawal/ Dependence (*n* = 12)	2 ± 1	2 ± 1
Energy/ Work Enhancement (*n* = 8)	4 ± 1	5 ± 1
Appetite Suppression (*n* = 5)	2 ± 1	3 ± 1
Social mood Enhancement (*n* = 6)	3 ± 1	3 ± 1
Physical Performance Enhancement (*n* = 3)	3 ± 1	4 ± 1
Anxiety/ Negative Physical Effect (*n* = 9)	2 ± 1	2 ± 1
Sleep Disturbance (*n* = 4)	3 ± 1	4 ± 1
Total Questions (*n* = 47)		

Note: Values are represented as means ± SD, *n* = number of questions.

**Table 2:** Acute effect of caffeine treatment (3 mg·kg^−1^) pre and post-7-week of resistance training on concentric and eccentric PT (Nm) of the elbow and knee flexors and extensors.

		Caffeine Intervention Group				Placebo Intervention Group			
	Angular Velocity (Deg/s)	Acute Placebo		Acute Caffeine		Acute Placebo		Acute Caffeine	
		Pre-Int	Post-Int	Pre-Int	Post-Int	Pre-Int	Post-Int	Pre-Int	Post-Int
Elbow Extension									
Con	60	38 ± 8	42 ± 8	41 ± 12	40 ± 7	36 ± 10	41 ± 11	42 ± 14	42 ± 10
	120	32 ± 8	38 ± 9	38 ± 14	35 ± 6	30 ± 9	35 ± 11	36 ± 14	38 ± 8
	180	30 ± 6	32 ± 7	33 ± 15	32 ± 6	30 ± 9	32 ± 8	32 ± 8	36 ± 8
Ecc	60	44 ± 11	48 ± 9	50 ± 16	46 ± 10	46 ± 18	48 ± 15	51 ± 17	49 ± 17
	120	40 ± 10 *	46 ± 8 ^^^	56 ± 19 *	48 ± 8 ^^^	41 ± 22 *	49 ± 13 ^^^	52 ± 13 *	54 ± 25 ^^^
	180	40 ± 10 *	49 ± 10 ^^^	59 ± 20 *	50 ± 11 ^^^	44 ± 28 *	51 ± 14 ^^^	55 ± 16 *	62 ± 27 ^^^
Elbow Flexion									
Con	60	51 ± 7 *^#^	56 ± 10 ^^#^	58 ± 16 *^#^	56 ± 9 ^^#^	51 ± 10	56 ± 15	60 ± 16 ^#^	61 ± 18 ^#^
	120	50 ± 10 *^#^	51 ± 9 ^^#^	52 ± 15 *^#^	53 ± 10 ^^#^	45 ± 11	54 ± 16	55 ± 15 ^#^	58 ± 11 ^#^
	180	45 ± 8 ^#^	48 ± 7 ^^#^	48 ± 15 ^#^	49 ± 9 ^#^	50 ± 12	49 ± 14	41 ± 11 ^#^	50 ± 10 ^#^
Ecc	60	54 ± 15	59 ± 12	61 ± 15	63 ± 21	62 ± 17	68 ± 22	64 ± 16	67 ± 21
	120	61 ± 17	59 ± 12	61 ± 15	63 ± 16	59 ± 13	68 ± 20	68 ± 19	70 ± 19
	180	61 ± 14	63 ± 10	61 ± 14	66 ± 14	65 ± 11	70 ± 17	62 ± 20	72 ± 19
Knee Extension									
Con	60	147 ± 47 ^#^	181 ± 67 ^#^	167 ± 61	188 ± 69	179 ± 59	187 ± 49	190 ± 41	175 ± 41
	120	139 ± 49 ^#^	160 ± 56 ^#^	150 ± 50	177 ± 62	153 ± 70	159 ± 65	166 ± 51	149 ± 41
	180	121 ± 46 ^#^	142 ± 52 ^#^	126 ± 58	141 ± 49	140 ± 54	122 ± 37	146 ± 63	126 ± 48
Ecc	60	156 ± 56	187 ± 66	187 ± 76	203 ± 73	192 ± 74	211 ± 72	212 ± 74	205 ± 52
	120	167 ± 54	174 ± 50	192 ± 75	221 ± 69	207 ± 76	208 ± 64	227 ± 88	189 ± 49
	180	161 ± 72	183 ± 46	202 ± 61	206 ± 60	225 ± 82	200 ± 61	230 ± 89	200 ± 52
Knee Flexion									
Con	60	92 ± 33 ^#^	112 ± 37 ^#^	103 ± 31	124 ± 38	111 ± 44 ^#^	115 ± 45 ^#^	119 ± 42	120 ± 45
	120	90 ± 32 ^#^	106 ± 33 ^#^	100 ± 37	119 ± 21	100 ± 40 ^#^	107 ± 42 ^#^	100 ± 35	100 ± 36
	180	80 ± 27 ^#^	96 ± 26 ^#^	89 ± 34	105 ± 25	87 ± 36 ^#^	97 ± 32 ^#^	97 ± 34	97 ± 32
Ecc	60	99 ± 37	125 ± 40	115 ± 36	135 ± 52	125 ± 47	140 ± 56	132 ± 45	142 ± 46
	120	98 ± 40	126 ± 46	121 ± 40	145 ± 37	125 ± 42	130 ± 50	119 ± 44	137 ± 50
	180	92 ± 27	119 ± 39	113 ± 30	142 ± 37	120 ± 43	128 ± 40	122 ± 40	138 ± 54

Note: Values are represented as means ± SD, PT = Peak Torque, Con = Concentric, Ecc = Eccentric, Pre-Int= Pre-training intervention, and Post-Int= Post-training intervention, matching * represents a significant effect of caffeine prior to the intervention, matching ^^^ represents a significant effect of caffeine post-intervention, matching ^#^ represents a significant difference on peak torque post the intervention.

**Table 3:** Acute effect of caffeine treatment (3 mg·kg^−1^) pre and post-7-week resistance training on concentric and eccentric TW (Nm) of the elbow and knee flexors and extensors.

		Caffeine Intervention Group				Placebo Intervention Group			
	Set	Acute Placebo		Acute Caffeine		Acute Placebo		Acute Caffeine	
		Pre-Int	Post-Int	Pre-Int	Post-Int	Pre-Int	Post-Int	Pre-Int	Post-Int
Elbow Extension									
Con	Set 1	393 ± 168	370 ± 66	348 ± 75	411 ± 228	344 ± 87	349 ± 105	369 ± 137	356 ± 66
	Set 2	298 ± 140	281 ± 63	266 ± 60	328 ± 230	277 ± 86	278 ± 63	311 ± 124	314 ± 85
	Set 3	232 ± 138	216 ± 53	216 ± 74	268 ± 213	213 ± 78	220 ± 61	246 ± 112	262 ± 98
Ecc	Set 1	428 ± 309	381 ± 91	410 ± 118	421 ± 193	366 ± 202	398 ± 132	444 ± 167	425 ± 141
	Set 2	334 ± 253	313 ± 72	334 ± 94	339 ± 225	303 ± 161	336 ± 108	478 ± 533	346 ± 131
	Set 3	279 ± 266	248 ± 52	265 ± 67	295 ± 208	237 ± 127	278 ± 104	303 ± 130	280 ± 95
Elbow Flexion									
Con	Set 1	486 ± 116	458 ± 91	490 ± 102	463 ± 71	500 ± 119	463 ± 107	523 ± 129	478 ± 92
	Set 2	377 ± 84	361 ± 70	369 ± 90	360 ± 82	395 ± 77	383 ± 104	412 ± 68	403 ± 76
	Set 3	312 ± 69	319 ± 78	321 ± 87	315 ± 71	341 ± 67	325 ± 68	362 ± 83	354 ± 80
Ecc	Set 1	538 ± 166 *	463 ± 87 ^^^	551 ± 134 *	505 ± 131 ^^^	493 ± 187 *	521 ± 205 ^^^	587 ± 194 *	537 ± 156 ^^^
	Set 2	416 ± 149 *	376 ± 80 ^^^	431 ± 118 *	402 ± 126 ^^^	408 ± 136 *	453 ± 149 ^^^	472 ± 133 *	448 ± 97 ^^^
	Set 3	345 ± 116 *	352 ± 89 ^^^	373 ± 85 *	345 ± 95 ^^^	372 ± 114 *	389 ± 104 ^^^	425 ± 135 *	409 ± 122 ^^^
Knee Extension									
Con	Set 1	612 ± 359 *^#^	1010 ± 361 ^#^	1080 ± 375 *	1045 ± 374	648 ± 402 *^#^	1036 ± 250 ^#^	981 ± 304 *	1120 ± 279
	Set 2	553 ± 342 *^#^	980 ± 351 ^#^	989 ± 278 *	971 ± 296	603 ± 403 *^#^	1006 ± 237 ^#^	1013 ± 164 *	1003 ± 189
	Set 3	495 ± 325 *^#^	856 ± 250 ^#^	911 ± 265 *	893 ± 252	598 ± 454 *^#^	903 ± 169 ^#^	967 ± 206 *	915 ± 124
Ecc	Set 1	586 ± 267	1036 ± 383	1179 ± 417	1144 ± 466	738 ± 407	1251 ± 423	1223 ± 401	1246 ± 275
	Set 2	503 ± 261	1011 ± 353	1084 ± 351	1061 ± 453	552 ± 345	1238 ± 411	1213 ± 320	1176 ± 253
	Set 3	435 ± 216	918 ± 301	990 ± 314	940 ± 368	572 ± 334	1171 ± 375	1110 ± 370	1146 ± 304
Knee Flexion									
Con	Set 1	524 ± 99	574 ± 179	569 ± 179	644 ± 169	547 ± 152	542 ± 127	563 ± 144	542 ± 144
	Set 2	442 ± 114	521 ± 173	485 ± 137	568 ± 104	448 ± 115	449 ± 96	477 ± 132	492 ± 150
	Set 3	404 ± 116	467 ± 140	476 ± 125	503 ± 97	379 ± 109	421 ± 110	450 ± 120	441 ± 108
Ecc	Set 1	505 ± 178 *	630 ± 241 ^^^	601 ± 235 *	699 ± 261 ^^^	610 ± 214 *	657 ± 210 ^^^	668 ± 235 *	630 ± 166 ^^^
	Set 2	496 ± 268	537 ± 200	528 ± 172	610 ± 220	514 ± 168 *	542 ± 165 ^^^	589 ± 213 *	578 ± 173 ^^^
	Set 3	422 ± 214	516 ± 193	482 ± 163	561 ± 203	430 ± 112 *	536 ± 182 ^^^	551 ± 199 *	545 ± 126 ^^^

Note: Values are represented as means ± SD, TW = Total Work, Con = Concentric, Ecc = Eccentric, Pre-Int= Pre-training intervention, and Post-Int= Post-training intervention, matching * represents a significant effect of caffeine prior to the intervention, matching ^^^ represents a significant effect of caffeine post-intervention, matching ^#^ represents a significant difference on total work post the intervention.

**Table 4:** Acute effect of caffeine treatment (3 mg·kg^−1^) pre and post-7-week resistance training on RPE following repeated MVC.

		Caffeine Intervention Group				Placebo Intervention Group			
		Acute Placebo		Acute Caffeine		Acute Placebo		Acute Caffeine	
		Pre-Int	Post-Int	Pre-Int	Post-Int	Pre-Int	Post-Int	Pre-Int	Post-Int
Elbow Extension	Set1	14 ± 3	16 ± 2	13 ± 2	16 ± 2	14 ± 2	14 ± 3	14 ± 2	15 ± 2
	Set2	15 ± 2	17 ± 2	15 ± 2	17 ± 2	15 ± 2	15 ± 3 ^^^	15 ± 2	17 ± 1 ^^^
	Set3	17 ± 2	19 ± 2	16 ± 2	19 ± 1	17 ± 1	16 ± 3 ^^^	17 ± 2	18 ± 2 ^^^
Elbow Flexion	Set1	14 ± 3	16 ± 2	14 ± 2	16 ± 2	14 ± 2	17 ± 2	14 ± 2	16 ± 2
	Set2	15 ± 2	18 ± 2	16 ± 2	17 ± 2	16 ± 2	18 ± 1	15 ± 2	18 ± 1
	Set3	17 ± 2	19 ± 2	17 ± 2	18 ± 2	17 ± 1	19 ± 1	17 ± 2	19 ± 1
Knee Extension	Set1	13 ± 3	17 ± 2	14 ± 2	16 ± 3	13 ± 3	16 ± 2	14 ± 2	16 ± 2
	Set2	14 ± 3	18 ± 2	15 ± 2	17 ± 3	13 ± 3	18 ± 1	16 ± 2	18 ± 1
	Set3	15 ± 2	19 ± 2	17 ± 2	18 ± 3	14 ± 3	19 ± 1	17 ± 2	19 ± 1
Knee Flexion	Set1	14 ± 2	16 ± 1	14 ± 2	16 ± 2	14 ± 3	16 ± 2	14 ± 2	16 ± 3
	Set2	15 ± 2	17 ± 2	15 ± 2	17 ± 2	15 ± 2	17 ± 1	16 ± 2	17 ± 2
	Set3	17 ± 2	19 ± 2	17 ± 2	18 ± 2	17 ± 2	19 ± 1	17 ± 2	19 ± 1

Note: Values are represented as means ± SD, RPE = Rate of Perceived Exertion, MVC = Maximum voluntary contraction, Pre-Int = Pre-training intervention, and Post-Int = Post-training intervention, matching ^^^ represents a significant effect of caffeine post-intervention.

## Data Availability

Not applicable.

## Supplementary Materials

The following are available online at https://www.mdpi.com/article/10.3390/nu13103367/s1, Table S1: Acute effect of caffeine treatment (3 mg·kg^−1^) pre-and post-7-week resistance training programme on readiness to invest effort physically, readiness to invest effort mentally, and felt arousal scale; Table S2. Effect of caffeine treatment (3 mg·kg^−1^) during the 7-week resistance training programme on sessional RPE.
