# Supplementary material for: Can 3 mg·kg−1 of Caffeine Be Used as An Effective Nutritional Supplement to Enhance the Effects of Resistance Training in Rugby Union Players?
Source: Nutrients. 2021 Sep 25;13(10):3367. doi: 10.3390/nu13103367 (PMC8539282; doi:10.3390/nu13103367)
Supplement: Supplementary file 1 [file nutrients-13-03367-s001.zip › nutrients-1377309-supplementary.pdf]

Supplementary Data S1

Table S1 Acute effect of caffeine treatment (3 mg·kg-1) pre-and post-7-week resistance training programme on readiness to invest effort physically, readiness to invest effort mentally, and felt arousal scale.

		Caffeine Group				Placebo Group			
		Placebo		Caffeine		Placebo		Caffeine	
	Time (min)	Pre	Post	Pre	Post	Pre	Post	Pre	Post
RTE Physical	Pre	5.3±2.3	5.5±2.6	6.1±3.1	5.8±2.8	5.9±2.6	4.7±2.4	5.8±2.7	4.5±2.7
	15	5.8±2.4	6.2±2.3	6.6±2.9	6.0±2.5	6.5±2.4	5.3±2.2	6.7±2.1	5.1±2.7
	30	6.1±2.4	6.3±2.2	6.9±2.7	6.6±2.2	6.7±2.4	6.0±1.5	7.1±2.0	5.5±2.6
	45	6.4±2.5	6.5±2.3	7.2±2.6	6.9±2.4	7.1±2.1	5.8±1.9	7.1±2.3	5.7±2.4
RTE Mental	Pre	5.6±1.9	5.4±2.5	5.8±2.7	5.6±3.0	5.6±2.7	4.6±2.5	5.9±2.7	4.6±2.1
	15	5.9±1.6	5.6±2.2	6.1±2.2	5.7±2.5	6.2±2.1	4.7±2.1	6.9±2.1	5.1±2.3
	30	6.5±1.9	6.0±2.1	6.4±2.4	7.5±3.6	6.9±2.3	5.5±2.0	7.0±2.1	5.5±2.3
	45	6.5±2.1	9.1±11.8	6.9±2.3	6.5±2.3	7.4±2.0	5.5±1.9	7.1±2.4	6.1±2.3
FAS	Pre	2±1	3±1	3±1	3±1	3±1	2±1	3±1	3±1
	15	3±1	3±1	3±1	3±1	3±1	3±1	3±1	3±1
	30	3±1	3±1	3±1	3±1	3±1	3±1	4±1	4±1
	45	4±1	3±1	4±1	4±1	4±1	3±1	4±1	4±1

Note: Values are represented as means ± SD, RTE = Readiness to Invest Effort, FAS = Felt Arousal Scale, Pre = pre-ingestion, and Post= post-ingestion.

Supplementary Data S2

Table S2 Effect of caffeine treatment (3 mg·kg-1) during the 7-week resistance training programme on sessional RPE.

		CP		SSP		SQ		DL		PC		HC	
		PL	CF	PL	CF	PL	CF	PL	CF	PL	CF	PL	CF
Session 1	Set 1	16±3	16±2	17±1	15±5	17±1	17±2	17±2	16±2	17±2	17±1	17±2	16±2
	Set 2	18±3	17±2	16±5	18±1	18±1	18±2	18±1	18±2	18±1	17±2	18±1	18±2
Session 2	Set 1	16±3	16±1	17±1	16±2	16±1	16±1	16±1	16±1	17±2	17±1	17±1	16±2
	Set 2	17±4	17±2	16±5	18±1	18±1	17±2	17±1	16±5	17±2	18±1	18±1	18±1
Session 3	Set 1	16±3	17±1	17±2	17±1	17±2	17±1	17±2	17±1	17±1	17±1	17±2	17±1
	Set 2	16±4	18±0	18±1	15±8	18±0	18±1	18±1	18±0	17±2	18±0	18±0	18±1
Session 4	Set 1	16±3	16±1	17±1	17±1	17±1	16±2	17±1	17±2	17±1	17±1	17±1	17±1
	Set 2	16±4	17±1	15±7	18±1	18±1	17±1	19±1	18±0	18±1	18±1	18±1	17±1
Session 5	Set 1	16±4	17±1	17±1	17±1	18±1	17±1	18±1	17±1	17±2	17±1	17±1	16±2
	Set 2	18±3	18±1	18±1	18±0	18±1	18±1	18±1	18±1	17±1	18±0	18±1	17±2
Session 6	Set 1	16±3	17±1	17±2	17±2	17±2	17±1	18±1	17±1	17±1	17±1	17±1	17±1
	Set 2	17±3	17±2	17±5	18±1	18±1	18±1	19±1	18±1	17±2	17±2	17±6	18±1
Session 7	Set 1	17±2	17±1	17±2	16±1	17±1	17±1	17±1	17±1	17±1	17±1	17±1	17±1
	Set 2	17±2	18±1	18±1	18±1	18±1	18±1	18±1	18±1	18±1	18±1	18±1	16±6
Session 8	Set 1	16±3	17±1	18±1	17±1	17±2	16±2	18±1	17±1	18±1	17±1	17±2	17±1
	Set 2	17±3	18±1	18±1	18±1	18±1	17±1	18±1	18±0	18±1	18±1	18±1	18±1
Session 9	Set 1	17±2	17±1	18±1	17±1	17±1	17±1	17±1	17±1	17±1	17±1	17±1	17±1
	Set 2	17±3	18±1	16±5	18±0	18±1	18±1	18±1	18±1	18±1	17±1	18±1	18±1
Session 10	Set 1	16±3	17±1	17±1	17±1	17±1	17±1	17±1	17±2	17±1	17±1	18±1	17±1
	Set 2	17±3	18±0	18±1	18±1	18±1	18±2	18±1	18±2	18±1	18±1	18±1	18±1
Session 11	Set 1	17±3	17±1	17±1	17±1	17±1	17±1	18±1	17±1	17±1	17±1	18±1	17±1
	Set 2	19±2	18±1	18±1	18±1	18±1	17±1	18±1	18±2	18±1	18±1	19±1	16±5
Session 12	Set 1	17±3	17±1	17±1	17±1	17±1	17±1	17±2	18±2	17±1	17±1	17±1	17±1
	Set 2	17±3	16±5	18±1	17±6	18±1	18±1	17±2	18±1	17±1	17±5	16±6	18±1
	Set 1	15±3	18±1	18±1	17±1	16±5	17±1	18±1	18±1	18±1	18±1	18±1	17±1

Session 13	Set 2	18±2	16±6	18±1	18±1	18±1	18±1	19±1	18±1	18±1	18±1	19±1	18±1
Session 14	Set 1	16±3	17±1	18±1	17±1	18±1	17±1	18±1	17±1	18±1	17±2	18±1	17±1
	Set 2	17±3	18±1	18±1	18±1	18±1	18±1	18±1	17±1	18±1	18±1	18±1	18±1

Note: Values are represented as means ± SD, RPE= Rating of Perceived Exhaustion, PL = Placebo Group, CF = Caffeine Group, CP= Chest Press, SSP= Seated Shoulder Press, SQ= Squats, DL = Deadlift, PC = Power Clean, HC = Hang Clean.
